# Urinary Measurement of Neutrophil Gelatinase Associated Lipocalin and Kidney Injury Molecule-1 Helps Diagnose Acute Pyelonephritis in a Preclinical Model

**DOI:** 10.1155/2013/413853

**Published:** 2013-12-14

**Authors:** Hahn-Ey Lee, Sun Hee Lee, Minki Baek, Hwang Choi, Kwanjin Park

**Affiliations:** ^1^Department of Urology, Seoul National University Hospital, 101 Daehak-no, Jongno-gu, Seoul 110-744, Republic of Korea; ^2^Department of Urology, Sungkyunkwan University, 81 Irwon-Ro Gangnam-gu, Seoul 135-710, Republic of Korea; ^3^Department of Urology, Armed Forces Capital Hospital, 177 Road, Bundang-gu, Sungnam si, Kyonggi do 463-040, Republic of Korea; ^4^Department of Urology, Seoul National University Children's Hospital, 101 Daehak-no, Jongno-gu, Seoul 110-744, Republic of Korea

## Abstract

*Background*. The study assessed whether measurement of urinary biomarkers of acute kidney injury could be helpful in diagnosing acute pyelonephritis and subsequent scarring. *Method*. *Escherichia coli* J96 (0.3 mL inoculum containing 1 × 10^9^/mL) was directly injected into the renal cortex of 3-week-old female Sprague Dawley rats (*n* = 20), with saline substituted in a control group (*n* = 10). Following the injection, urine was collected 2, 7, 14, 28, and 42 days after injection. Urinary neutrophil gelatinase associated lipocalin (NGAL), kidney injury molecule-1 (Kim-1), and interleukin-18 were quantitatively measured using enzyme-linked immunosorbent assay (ELISA). The levels of the biomarkers were adjusted for creatinine. Time course changes within a group or between the groups were compared. Correlation analysis was performed to understand the relationship between urinary levels and histological scarring. *Results*. Significantly elevated urinary NGAL was evident at two and seven days after injection, and Kim-1 was elevated at two days after injection. Receiver operating characteristic analyses confirmed the sensitivity of these markers at these times. No urinary marker at acute stage of APN was correlated with the amount of future scarring, negating their predictive value. *Conclusion*. Urinary NGAL and Kim-1 could be helpful in diagnosing febrile urinary tract infection in children.

## 1. Introduction

Febrile urinary tract infection (fUTI) has been reported in 1-2% of boys and 3–7% of girls younger than 6 years of age [1, 2]. Early diagnosis and treatment of fUTI are important, because missed or delayed diagnosis may result in the failure of appropriate treatment and possibly lead to long-term consequences, including renal scarring, hypertension, and chronic renal failure [3–5].

Unfortunately, the early and accurate diagnosis of fUTI is often challenging in children because of the lack of localizing signs and symptoms, difficulty in urine collection, and higher risks of contaminated samples. Although urine culture remains as the gold standard for diagnosing UTI [6], instant diagnosis is impossible due to the required incubation period of at least 24 h or more and another 2-3 days for complete identification of the bacteria, reducing the performance of this test in managing acutely sick children. Additionally, urine tests and imaging have unacceptable sensitivity and specificity in diagnosing fUTI. Hence, there is a clear need for a noninvasive, rapid, and highly sensitive test that will facilitate the early diagnosis of fUTI. Sensitive urine tests of potential biomarkers of fUTI are likely to obtain these goals.

fUTI occurs when adhesion of uropathogenic bacteria to renal parenchyme elicits acute pyelonephritis (APN) through the interaction between specific virulence factors and host immune response [7]. Given the wide variety of uropathogens and their specific virulence factors, it appears that markers related to host immune reaction may be a more appropriate target than bacteria related factors in detecting fUTI in humans. Also, these targets are expected to help us predict the development of future scarring, a likely result of host immune action rather than bacteria per se.

Recently, a series of biomarkers reflecting acute kidney injury (AKI) prior to the elevation of serum creatinine were identified [8]. Some also predicted future fibrosis as a result of AKI. Given that APN is a nonspecific renal injury, leading to consequent scarring as a result of tubulointerstitial fibrosis, we assumed that some biomarkers reflecting AKI process might reveal the presence of APN and help to predict future renal fibrosis. Based on a literature review, we chose three biomarkers of AKI as possible biomarkers of APN: neutrophil gelatinase associated lipocalin (NGAL), kidney injury molecule-1 (Kim-1), and interleukin-18 (IL-18). In this preclinical study, we tested whether the changes of these three urinary markers were related to acute renal parenchymal infection as well as chronic renal scarring in a rat model.

## 2. Methods

### 2.1. Animals

Thirty inbred 3-week-old female Wister rats weighing 80 gm were divided into test (*n* = 20) and control (*n* = 10) groups. We believed that these young-aged, immature rats might reflect a group of children who were susceptible to bacterial infection predisposing to renal scarring. The animals were housed in specific pathogen-free conditions at room temperature and received a typical rat diet with free access to tap water. These conditions satisfied the animal care guidelines of the Education and Research Center of Animal Models for Human Diseases, Seoul National University Hospital.

### 2.2. Bacteria

*Escherichia coli* strain J96 isolated from human patients with pyelonephritis was purchased from American Type Culture Collection (700336; ATCC, Manassas, VA, USA). It is motile, hemolytic, and positive for colicin V. A stock culture was grown overnight (10–12 h) on blood agar plates at 35°C. A single colony was inoculated onto LB broth and grown at 37°C with shaking to stationary phase. The bacteria were recovered by centrifugation and washed twice in phosphate-buffered saline. A solution containing approximately 10^9^ colony forming units (CFU) per milliliter was prepared.

### 2.3. Experimental Procedures

Pyelonephritis was induced as reported [7, 9]. Briefly, animals were anesthetized by intraperitoneal injection of Zoletil (zolazepam plus tiletamine) and Xylazine. The kidneys were exposed using a midline abdominal incision. Fifty microliters of a 10^9^ cfu/mL suspension of *E. coli* J96 was injected directly into the outer renal cortex of both the upper and lower poles of the left kidney using a 26-gauge needle. Control rats received isotonic saline only. Antibiotic treatment was delayed until 3 days of injection to facilitate the development of renal scarring. Urine was collected 2, 7, 14, 28, and 42 days after injection. Each urine sample was stored in aliquots at −80°C. Test and control kidneys were harvested 42 days after injection. Kidneys were bivalved; half was formalin fixed and paraffin embedded. Sections 4 *μ*m in thickness were cut, mounted on a slide, and stained with hematoxylin and Masson's trichrome to determine the tubular damage (dilation and atrophy) and interstitial fibrosis, respectively. Two independent observers blinded to the treatment group examined the slides microscopically to grade the severity of tubular damage and calculate the area of fibrosis. Tubular damage apparent as tubular dilation or atrophy was graded from 0 to 3 (0, no scarring; 1, <5% tubular damage; 2, 5–20% tubular damage; and 3, >20% tubular damage). Likewise, the area of renal fibrosis stained by Masson's trichrome as a percent to total area was determined by image analysis.

### 2.4. Enzyme-Linked Immunosorbent Assay (ELISA)

Direct ELISA was performed to detect urinary NGAL, Kim-1, and IL-18 using commercial kits. Urine samples were thawed approximately 1 h before the assays were performed. For all measurements, 100 *μ*L of diluted urine sample was analyzed in duplicate. For quantification, an 8-point standard curve was prepared by 1 : 3 dilution of a premixed standard containing all five analytes of a specific panel. The recommended dilution of 500-fold was optimal for the detection of NGAL, whereas no dilution was required for the detection of Kim-1 and IL-18. Urinary antigens were bound to the wells of microtiter plates by incubation of 100 *μ*L urine samples for 1 h at 37°C. Wells were blocked with buffer containing 5% bovine serum albumin. The primary antibody was goat polyclonal raised against rat NGAL (036; BioPorto, Grusbakken 8, Gentofte, Denmark), rat Kim-1 (E90785Ra; USCN Life Science, Houston, TX, USA), and rat IL-18 (E99064Ra; USCN Life Science, Wuhan, China). Incubation was followed by treatment with horseradish peroxidase-conjugated, affinity purified rabbit anti-goat IgG antibody. TMB substrate (BD Biosciences, Franklin Lakes, NJ, USA) was added for color development, which was read after 30 minutes at 450 nm with a Benchmark Plus microplate reader (Bio-Rad, CA, USA). The urinary creatinine (Cr) concentration was used to normalize NGAL measurements to account for the influence of urinary dilution on its concentration. Urinary levels of biomarkers were expressed as uNGAL/Cr ratio in ng/mg creatinine. Interassay and intraassay coefficient variations were 5% to 10% for batched samples analyzed on the same day.

### 2.5. Statistical Analysis

Time course changes of the three markers in the control and APN groups were plotted, and the level of significance of within or between groups was determined with repeated measure analysis of variance. Also, we evaluated whether the urinary level at acute phase of UTI (i.e., two or seven days after injection) could be helpful in diagnosis by plotting receiver operator characteristics (ROC) curves for each study using the Graph Pad Prism 5 software package (GraphPad Software, La Jolla, CA, USA). To assess whether the urinary level at acute APN could help to predict future scarring or fibrosis, correlation analysis was done between the urinary level at two and seven days following injection and the grade of tubular damage or the amount of tubulointerstitial fibrosis. All urinary levels were adjusted for creatinine (Cr). The tests were considered significant at a *P* value <0.05.

## 3. Results

### 3.1. Time Course Changes of Each Urinary Marker

**Figure 1:**
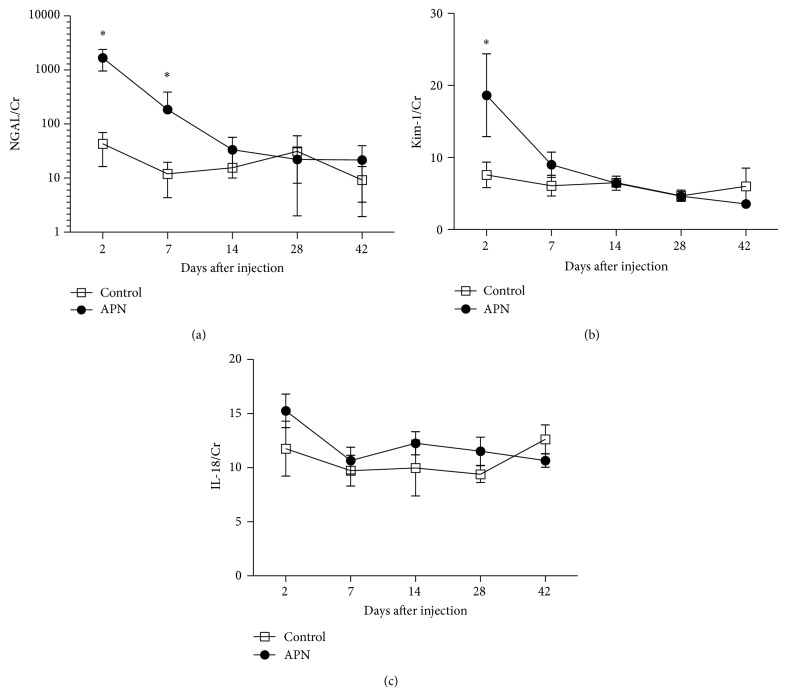
Time course changes of the three urinary biomarkers during the acute and chronic phase of APN. Compared to control (empty circles), significantly higher levels were noted in APN group (filled circles) at two and seven days of NGAL/Cr and two days of Kim-1/Cr. In contrast, no significant difference was noted in urinary IL-18/Cr during the period of study.

The temporal changes of the three biomarkers were compared between the APN and control groups (Figure 1). The mean urinary level of NGAL/Cr was significantly higher than that of control two and seven days after injection. The level declined thereafter to be comparable to the control group for the remaining period. Similarly, significant elevation of mean urinary level of Kim-1 was evident two days after injection, although the difference between the infection and control group was not as great as in the case of NGAL. Unlike the other markers, comparison of mean urinary level of IL-18 revealed no significant difference at any time point between the test and control groups.

### 3.2. ROC Analysis

**Figure 2:**
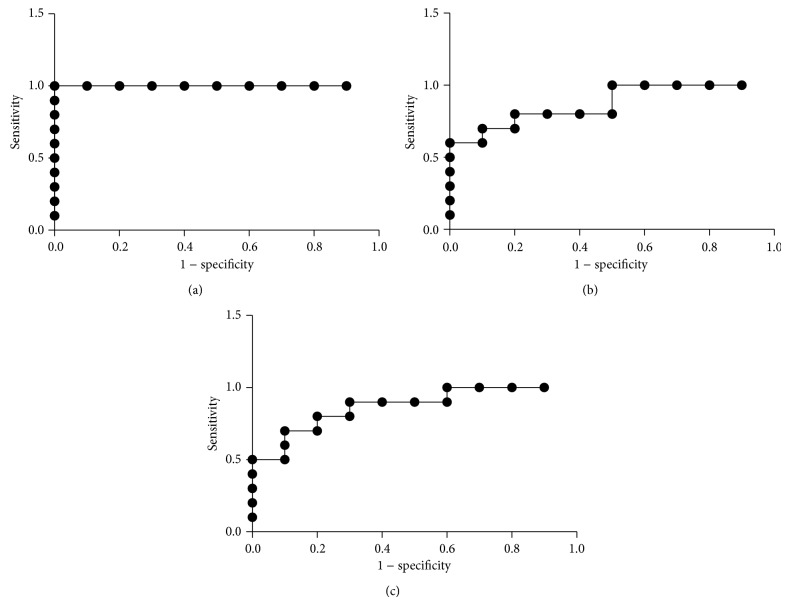
Receiver operating characteristics curve analysis for NGAL/Cr at two days (a) and seven days (b) and for Kim-1/Cr at two days (c) when a significantly higher urinary level was evident in the APN group compared to control. The area under curve was 1.0 (SE: 0.0, 95%, CI 1.0), 0.87 (SE: 0.08, 95%, CI 0.714–1.03), and 0.87 (SE: 0.08, 95%, CI 0.714–1.03) for NGAL/Cr at day two and seven and for Kim-1/Cr at two days, respectively.

ROC curves compared the performance of the urinary biomarkers in detecting acute renal infection, wherein the area under the ROC curve serves as a measure for the overall ability to discriminate between control and APN animals. The analysis revealed that urinary level of NGAL at days two and seven following injection was a sensitive marker, with the area under the curve (AUC) exceeding 0.5 (Figure 2). For urine samples collected two days after APN, using an optimal cutoff level of 306 ng/mg for NGAL/Cr for the diagnosis of APN, the sensitivity and specificity were both 1.0, revealing perfect diagnostic power. For the samples collected at seven days of APN, using an optimal cutoff level of 89.6 ng/mg for NGAL/Cr for the diagnosis of APN, sensitivity and specificity were both 0.8. The level of Kim-1 at day two could discriminate between the control and APN rats. For samples collected at two days of APN, using an optimal cutoff value of 11.7 ng/mg for Kim-1/Cr for the diagnosis of APN, sensitivity and specificity were both 0.8.

### 3.3. Correlation between Urinary Levels and the Degree of Scarring

To explore the feasibility of the markers in predicting the amount of scarring, correlation analysis was performed between the amount of scarring including tubular damage or tubulointerstitial fibrosis and urinary levels of each marker. No biomarker at any time point was correlated with the amounts of scarring (Table 1).

## 4. Discussion

In this study, three novel urinary biomarkers of acute renal injury were investigated in urine using commercial ELISA assays to test whether these markers could be used to detect acute renal parenchymal infection and to predict or measure subsequent scarring.

Both urinary NGAL and Kim-1 turned out to be eligible candidate biomarkers in detecting acute renal parenchymal infection. Significant elevations of uNGAL/Cr (two and seven days) and uKim-1/Cr (two days) occurred only in the acute phase of renal infection. The diagnostic value of these two markers was further supported by ROC analysis showing significant AUC (>0.8). This high AUC value suggests that detection of acute pyelonephritis may be aided by simply measuring the urine level of these biomarkers in cases of fever of unknown origin.

Only IL-18 was ineligible to detect APN. No elevation was found in uIL-18/Cr during the test period. Although IL-18 was reported to be a promising marker to predict acute renal injury prior to elevation of serum creatinine, it is probable that the marker was specific to ischemic renal injury rather than urinary tract infections as previously reported [10].

The elevation of uNGAL/Cr in APN has already been reported in a preclinical model [11]. However, our data featured different kinetics of uNGAL/Cr from that previously reported. In the earlier study, significant elevation of uNGAL/Cr was observed 1 week after APN and this elevation persisted for 6 weeks of APN. Thus, the previous authors stressed the value of NGAL as a marker of scarring rather than acute infection. While we confirmed their results showing the significant elevation of uNGAL/Cr after 1 week of APN, our data suggest that the elevation of uNGAL/Cr was more prominent at day two of infection rather than day seven, revealing a rise in uNGAL/Cr that was more rapid than previously reported. This is an evidence of the value of uNGAL/Cr as a diagnostic marker of APN rather than a prognostic marker for scarring.

The most contrasting difference from what was reported in the earlier study was the lack of significant elevation of NGAL after 7 days. The previous study authors attributed the secretion of NGAL by renal proximal tubule to the persistence of the elevated level. Considering the kinetics of NGAL showing acute elevation and rapid decline, we can assume that the source of NGAL elevation was mainly from neutrophils rather than cells in the proximal tubule. The reason for the discrepancy between studies may be the difference of susceptibility of rats and bacterial virulence causing difference in invoking NGAL expression in proximal tubule. In pediatric patients, a report described the rapid decline of serum level of NGAL within 2 days of bacterial infection [12]. Hence, our data might better correspond to the clinical situation.

The elevation of urinary NGAL or NGAL/Cr in UTI was reported in 60 patients in a clinical series of UTI [13]. However, the diagnostic value of NGAL in APN was not confirmed in this study because the majority (73%) of the patients turned out to have nonfebrile UTI, which did not involve the kidney (lower tract UTI). The clinical diagnostic value of NGAL for APN remains to be established.

Another novel finding of the present study was the elevation of uKim-1 during the acute phase of infection. Although the absolute difference from control and the amount of AUC in ROC curve analysis were not as great as in the case of NGAL, the fact that significant difference was only found at the very acute stage of APN (i.e., two days of APN) may suggest its greater specificity than NGAL in diagnosis of acute infection. Kim-1 is rarely expressed in normal kidney and markedly upregulated expression has been reported in response to proximal renal tubule injury [14]. Thus, APN involving tubulointerstitial region in the kidney could promote the expression and lead to increased urinary level. This potential benefit should be verified in clinical population in future study.

Another objective in this study was to assess the predictability of these urinary biomarkers for future renal scarring. Based on the potential role of these biomarkers in tubulointerstitial fibrosis and previous results that demonstrate the persistence of NGAL following APN [11], it was expected that these biomarkers could play a role in the development of scarring. Also, to understand which stage of scarring was affected by these markers, the level of each marker was correlated with either tubular damage or the amount of fibrosis. However, no marker was associated with either tubular damage or the amount of fibrosis after 6 weeks of APN. This was an unexpected finding. It is conceivable that the sample numbers were too few to draw any definite conclusion or the fact that we evaluated the scarring by microscopic histological assay on the sampled area rather than macroscopic DMSA for the whole kidney as has been done in a previous clinical study [15]. However, based on our data, we believe that the potential use of both NGAL and Kim-1 is likely to be valuable in diagnosis in acute APN rather than predicting future scarring.

## Figures and Tables

**Table 1:** Correlation analysis between each component of renal scarring (tubular changes and amount of fibrosis) and the levels of urinary biomarkers at early phase of APN.

	NGAL/Cr	NGAL/Cr	Kim-1/Cr	Kim-1/Cr	IL-18/Cr	IL-18/Cr
	at 2 days	at 7 days	at 2 days	at 7 days	at 2 days	at 7 days
Tubular damage						
Coefficients (*r*)	0.20	0.51	−0.01	0.16	0.24	−0.09
*P*	0.58	0.13	0.91	0.64	0.56	0.77
Amount of fibrosis						
Coefficients (*r*)	0.31	−0.48	0.25	0.11	0.18	0.26
*P*	0.39	0.16	0.59	0.72	0.45	0.75
